# The Acute Readiness Monitoring Scale: Assessing Predictive and Concurrent Validation

**DOI:** 10.3389/fpsyg.2021.738519

**Published:** 2021-09-24

**Authors:** Simon J. Summers, Richard J. Keegan, Andrew Flood, Kristy Martin, Andrew McKune, Ben Rattray

**Affiliations:** ^1^Discipline of Sport and Exercise Science, Faculty of Health, University of Canberra, Canberra, ACT, Australia; ^2^Brain Stimulation and Rehabilitation (BrainStAR) Lab, Western Sydney University, Penrith, NSW, Australia; ^3^Research School of Biology, Australian National University, Canberra, ACT, Australia; ^4^Research Institute for Sport and Exercise, University of Canberra, Canberra, ACT, Australia; ^5^Discipline of Psychology, Faculty of Health, University of Canberra, Canberra, ACT, Australia; ^6^School of Health Sciences, University of KwaZulu-Natal, Durban, South Africa

**Keywords:** readiness, cognitive performance, cortisol, heart rate variability, sleep deprivation

## Abstract

To complement and enhance readiness-monitoring capability, the Acute Readiness Monitoring Scale (ARMS) was developed: a widely applicable, simple psychometric measure of perceived readiness. While this tool may have widespread utility in sport and military settings, it remains unknown if the ARMS demonstrates predictive and concurrent validity. Here, we investigated whether the ARMS is: (1) responsive to an acute manipulation of readiness using sleep deprivation, (2) relates to biological markers of readiness [cortisol/heart-rate variability (HRV)], and (3) predicts performance on a cognitive task. Thirty young adults (aged 23 ± 4 years; 18 females) participated. All participants engaged in a 24-h sleep deprivation protocol. Participants completed the ARMS, biological measures of readiness (salivary cortisol, HRV), and cognitive performance measures (psychomotor vigilance task) before, immediately after, 24-, and 48-h post-sleep deprivation. All six of the ARMS subscales changed in response to sleep deprivation: scores on each subscale worsened (indicating reductions in perceived readiness) immediately after sleep deprivation, returning to baseline 24/48 h post. Lower perceived readiness was associated with reduced awakening responses in cortisol and predicted worse cognitive performance (slower reaction time). No relationship was observed between the ARMS and HRV, nor between any biological markers of readiness (cortisol/HRV) and cognitive performance. These data suggest that the ARMS may hold practical utility in detecting, or screening for, the wide range of deleterious effects caused by sleep deprivation; may constitute a quick, cheap, and easily interpreted alternative to biological measures of readiness; and may be used to monitor or mitigate potential underperformance on tasks requiring attention and vigilance.

## Introduction

The ability to measure an individual's readiness to perform in an upcoming task can have wide-ranging implications. For example, in a sporting or military context, identifying those ready to engage in an upcoming activity will likely impact sports performance (Ortega and Wang, 2018) or operational success (Szivak and Kraemer, 2015). While tools for the assessment of individual readiness exist in various contexts (e.g., salivary cortisol, heart rate variability), most involve time- and resource-intensive examinations (Richard et al., 2020) and lack the sophistication to appropriately account for what constitutes readiness (Saw et al., 2016). As such, an obvious need exists for a metric of readiness that can be easily deployed and allow for the investigation of readiness in a range of contexts. In response, the *Acute Readiness Monitoring Scale* (ARMS) was developed; a multidimensional, self-report tool of perceived readiness. The ARMS measures an individual's perceived readiness, defined as an acute state of preparation and capability to perform any key task or role, in the immediate future (Richard et al., 2020).

The ARMS was established in a military population and validated against existing measures of current affect and recent psychological distress (Richard et al., 2020). However, it remains unclear whether the ARMS: (a) is responsive to manipulations of readiness; (b) relates to other biological markers of readiness; and (c) predicts task performance. Understanding these aspects is essential, particularly if the ARMS is to have practical utility in monitoring readiness, well-being and capability in a broad range of contexts (e.g., athletic performance, Army deployment).

Sleep deprivation presents as one avenue to manipulate an individual's readiness to perform. Previous research has shown sleep deprivation to have a significant impact on cognitive and physical performance (Alhola and Polo-Kantola, 2007; Parker and Parker, 2017; Grandou et al., 2019). Specifically, reductions in vigilance have been reported in young healthy sleep deprived individuals (Roca et al., 2012), with similar cognitive decrements reported in elite athletic and military populations (Knufinke et al., 2018; Beckner et al., 2021). Impaired task performance and physical performance in military personnel has also been observed during sustained operations involving sleep deprivation (Nindl et al., 2002). While there are no gold standard markers of physiological stress, these findings suggest that sleep deprivation may be an ecologically valid and effective means through which to test the responsiveness of the ARMS to an acute manipulation of readiness. Therefore, the first aim of this study was to investigate the responsiveness of the ARMS to a 24-h sleep deprivation protocol.

Sleep deprivation also appears to produce consistent changes in stress-related biomarkers. In particular, heart rate variability (HRV) has been shown to decrease in response to acute sleep deprivation (Vaara et al., 2009; Bourdillon et al., 2021). Specifically, markers of parasympathetic activity such as the root mean square of the successive differences (RMSSD) and power in the high frequency (HF) band have been shown to decrease following 24-h of sleep deprivation (Chen et al., 2013; Morales et al., 2019). Importantly, these changes in HRV have been shown to predict the cognitive decrements associated with sleep deprivation (Chua et al., 2012; Gamble et al., 2018). Similarly, decreases in the cortisol awakening response is also observed in sleep deprived individuals (Vargas and Lopez-Duran, 2020). In the context of such findings, these biomarkers of readiness (HRV and cortisol) provide an opportunity to validate the ARMS against measures that have been reported to be sensitive to fluctuations in readiness resulting from sleep deprivation. Further, HRV and cortisol are commonly implemented biomarkers of the physiological stress response to sleep deprivation in athletic and military settings (Gabbett et al., 2017; Tomes et al., 2020). Therefore, the second aim of this study was to examine the relationship between the ARMS and biological markers of readiness including cortisol and HRV.

Although the ARMS was developed as a measure of perceived preparation and capability to perform, its predictive utility has not yet been assessed against individual task performance. Therefore, the third aim of this study was to investigate whether the ARMS correlates with performance on a cognitive task. Cognitive performance, specifically performance on a vigilance task, was selected based on the wide body of literature reporting decrements in performance of this task in response to sleep deprivation (Lara et al., 2014; Hudson et al., 2020). Further, cognitive performance in this context has particular relevance to sports and military job roles that require underlying capacities for sustained attention (e.g., operational/tactical personnel and pilots, and those sports that require intense concentration such as golf and motor sports) (Samuels, 2012; Szivak and Kraemer, 2015).

## Materials and Methods

### Participants

As this is the first study to investigate the responsiveness of the ARMS to sleep deprivation, no data exists to support a sample size calculation. As such, an exploratory investigation was conducted with 30 university students between 18 and 40 years of age [mean ± standard deviation (SD) age 23 ± 4 years; 18 females]. Thirty participants were chosen since similar sample sizes have been used in previous research investigating the responsiveness of other subjective scales (e.g., Karolinska Sleepiness Scale) to sleep deprivation (Kaida et al., 2006). Participants were excluded if they presented with a neurological condition, sleep disorder, mental illness or visual impairment that may affect their sleep, cognition or psychological response to stress. Night shift workers, and those that could not understand instructions or questions provided in English were also excluded from participation. All participants provided written informed consent before testing, and all procedures were approved by the local institutional Human Research Ethics Committee (4,683).

### Experimental Protocol

**Figure 1:**
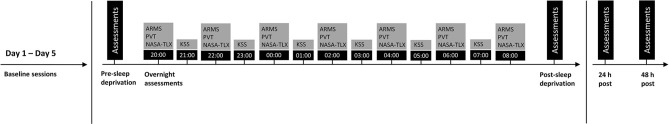
Experimental protocol. Assessments, including the Acute Readiness Monitoring Scale (ARMS), biological markers of readiness (heart rate variability, saliva sample) and cognitive performance [psychomotor vigilance test (PVT)] were taken before (pre-sleep deprivation) and after (post-sleep deprivation, 24- and 48 h post) sleep deprivation. Overnight assessments (from 20:00 to 08:00 h) included the ARMS, PVT, National Aeronautics and Space Administration Task Load Index (NASA-TLX) and Karolinska Sleepiness Scale (KSS). These were collected in order of presentation (from left to right, top to bottom).

All participants were involved in five consecutive days of data collection (Day 1–5, Figure 1), prior to engaging in a 24-h sleep deprivation protocol. The first 5 days of assessments were performed at home by each participant. Participants completed the ARMS and biological measures of readiness (HRV, salivary cortisol) prior to breakfast each morning, and completed the cognitive task [psychomotor vigilance task (PVT)] anytime between 9:00 and 12:00 h. Sleep (Actigraphy) and physical activity levels (exercise diary including type, duration, rate of perceived exertion per session) were monitored throughout these 5 days. Multiple days of baseline assessments were used to establish usual sleep and physical activity data and to ensure familiarisation in cognitive task performance prior to sleep deprivation (i.e., minimise carryover of training effects from repeated exposure to PVT).

All participants engaged in the sleep deprivation protocol within seven days of completing the baseline assessments. Twenty four-hour sleep deprivation protocols have been used extensively to understand the impact of fatigue on cognitive and physical performance (Joo et al., 2012; Trksak et al., 2013; Ghanbari et al., 2019; Ołpińska-Lischka et al., 2020). On the day of the sleep deprivation protocol, participants completed the ARMS and biological measures of readiness (HRV, saliva sample) at home, prior to breakfast. Participants then attended a laboratory session (between 9:00 and 12:00 h) where they completed the cognitive assessment (PVT) under the supervision of one of the researchers (pre-sleep deprivation measure, Figure 1). That evening, participants were instructed to stay awake, at home, from 20:00 to 8:00 h. Throughout this period, participants completed the ARMS and cognitive assessment (PVT) every 2 hours, starting from 20:00 h. The National Aeronautics and Space Administration Task Load Index (NASA-TLX) was also completed immediately after each PVT to assess subjective workload. Every alternate hour to the cognitive assessment, participants completed the Karolinska Sleepiness Scale (KSS) to measure subjective sleepiness. Following the last assessment (at 8:00 h), participants completed the ARMS and biological measures (HRV, saliva sample) again at home, before attending the laboratory to complete the cognitive assessment (PVT) (post-sleep deprivation measure, Figure 1). The scheduled time for the post-sleep deprivation lab session was matched to the scheduled time for the pre-sleep deprivation lab session for each participant. Participants were asked to avoid consumption of caffeine and alcohol, and from engaging in exercise throughout the night of the sleep deprivation protocol–which was checked using the actigraphy. All assessments were collected for another 2 days (24- and 48-h measure, Figure 1) by each participant at home. These outcomes were collected at the same scheduled times as those recorded immediately post-sleep deprivation.

### Assessments

All assessments were performed by a single researcher in the same laboratory (SJS).

### Acute Readiness Monitoring Scale

The ARMS is a 32-item scale that is designed to assess acute, multidimensional readiness in Army personnel. The questionnaire has nine factors of readiness: (1) Overall Readiness; (2) Physical Readiness; (3) Physical Fatigue; (4) Cognitive Readiness; (5) Cognitive Fatigue; (6) Threat-Challenge Readiness; (7) Group-Team Readiness; (8) Skills-Training Readiness; and (9) Equipment Readiness. Data from readiness factors 1–6 were used for analysis, while the remaining factors (7–9) were excluded, as these items were not relevant to the study aims and methods. In the original development of the ARMS (manuscript under review), content was informed by existing self-report measures of resilience, stress recovery, fatigue, and coping, as well as liaising with academic experts and relevant user groups (i.e., Army personnel). Exploratory and Confirmatory Factor Analysis was used to test the factor structure of the ARMS. The finalised questionnaire was then tested in a sample of 770 Australian Army personnel (male = 677, female = 93), demonstrating excellent internal consistency (Cronbach's alpha > 0.80). Concurrent and discriminant validity was also supported through comparisons to measures of theoretically related and unrelated constructs.

### Psychomotor Vigilance Task

The PVT is a valid assessment of an individual's level of vigilance and sustained attention and has been used extensively to evaluate the cognitive effects of sleep deprivation (de Bruin et al., 2017). During this task, participants sat in an upright chair ~60 cm from a laptop screen (15 inch; resolution of 1,366 × 768), with their index finger (of their dominant hand) resting on the spacebar key of the laptop. Participants were instructed to monitor a red rectangular box on the black background of the laptop screen for a yellow stimulus counter that appeared within the rectangle at random intervals (2,000–10,000 ms). As soon as the stimulus counter appeared, participants were instructed to press the spacebar key as quickly as possible, which stopped the counter and displayed the reaction time in milliseconds. Once the participants were familiarised with the task, they were given the study laptop to enable at home assessments of the PVT. Participants were instructed to perform at home assessments in a quiet, well-lit room, without distractions. This laptop was also used to administer the PVT during the laboratory sessions. The PVT was administered using the program E-Prime, with each session taking ~5 min to complete (40 trials). The mean reaction time (RT) across each session was used for analysis. Reaction times <100 ms (premature responses) and >500 ms (lapses in concentration) were excluded from the analysis (Basner and Dinges, 2011).

### Heart Rate Variability

Each participant was fitted with a Polar H10 heart rate sensor chest strap to assess HRV. The Polar H10 chest strap collected and processed HRV measurements by detecting the electrical signals of the heart. This method has been validated against electrocardiogram recordings (Gilgen-Ammann et al., 2019). Polar H10 chest straps were connected to the EliteHRV^©^ app (*via* Bluetooth 4.0 signal) that was downloaded on each participant's phone—as previously described (Speer et al., 2020). For each measurement, participants were required to lay resting, in supine, and collect a 5 min recording of their heart rate using the app. The raw R-R interval data of the 5 min recording was exported as a text file to Kubios HRV software (version 3.1.0, Biosignal Analysis and Medical Imaging Group, Kuopio, Finland) for analysis of HRV parameters within the time domain (root mean square of successive differences between normal heartbeats, RMSSD). This measure was then log transformed for analysis (Kobayashi et al., 2012). The RMSSD is a valid measure of HRV and has been shown to be responsive to sleep deprivation (Bourdillon et al., 2021).

### Salivary Cortisol

Two passive drool saliva samples were collected in sterilised cryovials, the first upon waking whilst still in bed and the second 30 min post-waking. All participants were instructed to avoid any food or fluid prior to providing the saliva samples. The participants received training on the saliva collection procedure prior to study commencement. They were requested to adhere as closely as possible to the procedure, which was carried out in their home. Participants recorded the time each saliva sample was collected. All samples were frozen immediately after collection in home freezers. Upon completion of the study, participants brought all saliva samples in a cooler bag to the laboratory, upon which they were stored at −20°C until analysis. Transport time outside the freezers is estimated to be between 5 and 20 min. Saliva was assayed for cortisol using commercial enzyme immunoassay kits (Salimetrics LLC, State College, PA). To reduce between-person variability, samples from the same participant were tested using the same analysis kit. The intra-assay coefficient of variation (CV) was 3.44% and the inter-assay CV was 6.23%. The awakening response in cortisol was used for analysis (i.e., the change in cortisol from the waking response to the 30 min post-measure) (Elder et al., 2014).

### National Aeronautics and Space Administration Task Load Index

The NASA-TLX is composed of six subscales: Mental Demand (“How mentally demanding was the task?”); Physical Demand (“how physically demanding was the task?”); Temporal Demand (“How hurried or rushed was the pace of the task?”); Performance (“How successful were you in accomplishing what you were asked to do?”); Effort (“How hard did you have to work to accomplish your level of performance?”); and Frustration (“How insecure, discouraged, irritated, stressed, and annoyed were you?”) (Hoonakker et al., 2011). Participants were asked to score each item on a scale, divided into 20 equal intervals anchored by the descriptors very high and very low. This score was multiplied by 5, resulting in a final score between 0 and 100 for each of the subscales. The NASA-TLX is a valid assessment of mental workload and has demonstrated good test-retest reliability (Xiao et al., 2005; Hart, 2006).

### Karolinksa Sleepiness Scale

The KSS was used to assess subjective sleepiness (Kaida et al., 2006). Participants were asked to rate their sleepiness on a 10-point scale (1 = extremely alert, 2 = very alert, 3 = alert, 4 = rather alert, 5 = neither alter nor sleepy, 6 = some signs of sleepiness, 7 = sleepy, but no effort to keep awake, 8 = sleepy, but some effort to keep awake, 9 = very sleepy, great effort to keep awake, fighting sleep, and 10 = extremely sleepy, can't keep awake). The reliability and validity of this scale has been demonstrated (Kaida et al., 2006).

### Statistical Analysis

Data were analysed using Statistical Package for the Social Sciences software (version 23 IBM Corp, Armonk, NY, USA) and R statistical package (version 3.4.2). To assess the responsiveness of the ARMS to sleep deprivation (Aim 1), linear mixed effect models (LMEM) were fitted to each ARMS subscale (overall readiness, physical readiness, physical fatigue, cognitive readiness, cognitive fatigue, threat-challenge readiness) with repeated measurements (pre-sleep deprivation, post-sleep deprivation, 24- and 48-h post-sleep deprivation) as the fixed effect and the participant as the random effect. The same statistical model was applied to assess the responsiveness of HRV, cortisol, and cognitive performance to sleep deprivation. For these analyses, data met assumptions of normality and homogeneity of variance as determined by Q–Q plots and the Mauchly's test, respectively. Where appropriate, *post-hoc* analyses were performed using Sidak-adjusted multiple comparison tests. To examine the relationship between the ARMS and biological markers of readiness (Aim 2), repeated-measures correlations were conducted between each ARMS subscale and HRV and cortisol. Repeated-measures correlations provided the within-individual association for measures assessed across all time points (pre-sleep deprivation, post-sleep deprivation, 24- and 48-h post-sleep deprivation). Unlike simple regression, repeated-measures correlations do not violate assumptions of independent observations, and tend to have greater statistical power as no data averaging or aggregation is required (Bakdash and Marusich, 2017). Similarly, repeated-measures correlations were used to examine the relationship between the ARMS subscales and cognitive performance (Aim 3). All correlations were interpreted by using the following values: <0.30 = weak; 0.30–0.49 = moderate; and ≥0.50 = strong association (Cohen et al., 2013). Due to the exploratory nature of this study, a Bonferroni correction was not applied for the multiple correlation analyses, as this was considered too conservative (Bender and Lange, 2001).

As an additional exploratory analysis to provide further support for any correlations identified between the ARMS and cognitive performance, multivariate linear regressions were conducted assessing the predictive value of the ARMS on cognitive performance. These regression models were performed with and without HRV and cortisol to determine if combining perceptual (ARMS) and biological indicators of readiness was more or less predictive of cognitive performance compared to perceptual measures alone. Model covariates (HRV/cortisol) were included if variance inflation factors were <2 and their correlations with one another were <0.4 (O'brien, 2007). Goodness-of-fit [Akaike information criterion (AIC) and Bayesian information criterion (BIC)] and r-squared values (conditional and marginal) were calculated for each model (Jaeger et al., 2017). All data are presented as mean and standard deviations. Statistical significance was set at *p* < 0.05.

## Results

### Participant Characteristics

Participant characteristics are summarised in Table 1. Sleep and exercise data were recorded for the first 5 days (baseline assessments). Participants were young active adults, exercising an average of three out of the 5 days, with an average duration of 63 min per/session. Participants engaged in a range of activities including, running, swimming, weights, and sporting activities (e.g., football). Average sleep time per night over the 5 day assessment was 385 min (6.4 h).

**Table 1:** Participant demographics.

	**Group mean (SD)**
Age (years)	23 (4)
Gender (males/females)	12/18
Height (cm)	167 (8)
Weight (kg)	70 (14)
Sleep time (min)	385 (19)
Number of exercise sessions	3 (2)
Duration of exercise per session (min)	63 (range: 15–240)
RPE of exercise per/session	5 (2)

*SD, standard deviation; RPE, rate of perceived exertion; kg, kilogrammes; cm, centimetres*.

### Overnight Assessments During Sleep Deprivation

**Figure 2:**
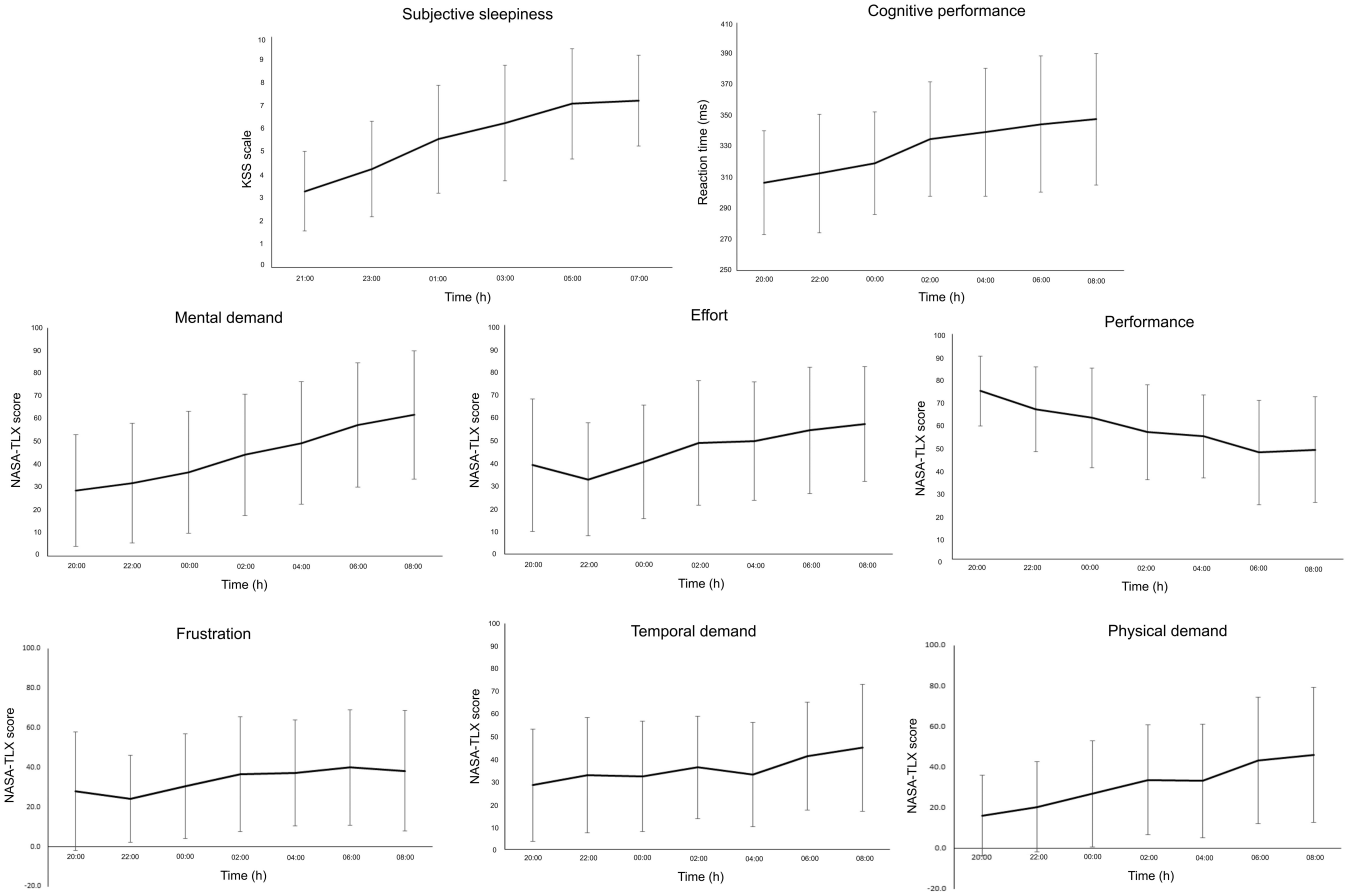
Mean and standard deviation of subjective sleepiness [Karolinksa Sleepiness Scale (KSS)], cognitive performance (psychomotor vigilance task), and subjective workload [National Aeronautics and Space Administration Task Load Index (NASA-TLX)] for each subscale (Mental Demand, Effort, Performance, Frustration, Temporal Demand, Physical Demand) during the overnight assessments (from 20:00 to 08:00 h).

**Figure 3:**
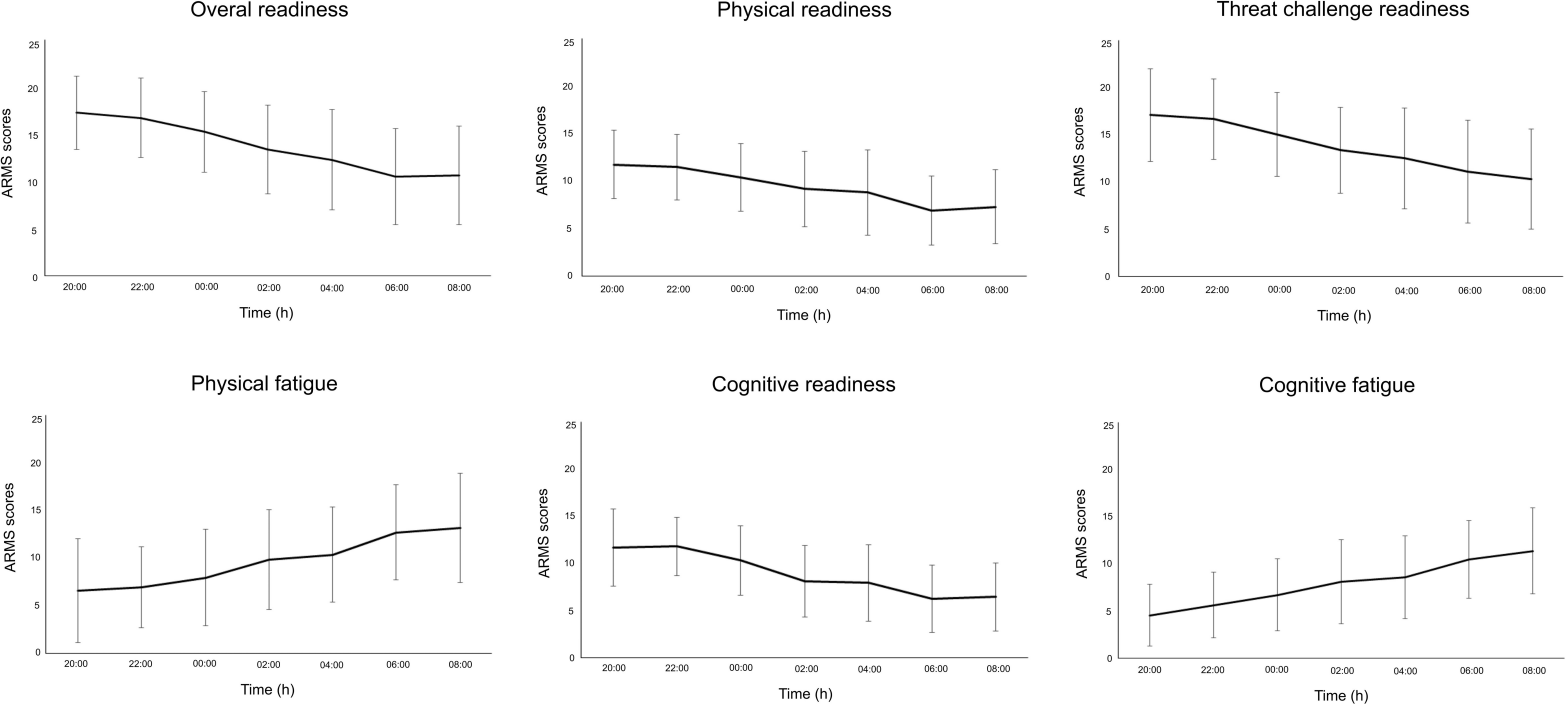
Mean and standard deviation for each subscale of the Acute Readiness Monitoring Scale (ARMS) during the overnight assessments (from 20:00 to 08:00 h).

Participants reported increased sleepiness throughout the night (Figure 2). This paralleled the trend in cognitive performance and subjective workload—that is, progressively slower reaction time (PVT task) and increased workload scores (NASA-TLX) (Figure 2). All subscales of the ARMS appeared to progressively decline throughout the night: Overall Readiness, Physical Readiness, Cognitive Readiness, and Threat Challenge Readiness reduced, while Physical Fatigue and Cognitive Fatigue increased (Figure 3) (statistical comparison of the ARMS before, immediately after, and 24- and 48 h post-sleep deprivation is presented below).

### The Responsiveness of the ARMS, HRV, Cortisol, and Cognitive Performance to Sleep Deprivation

**Figure 4:**
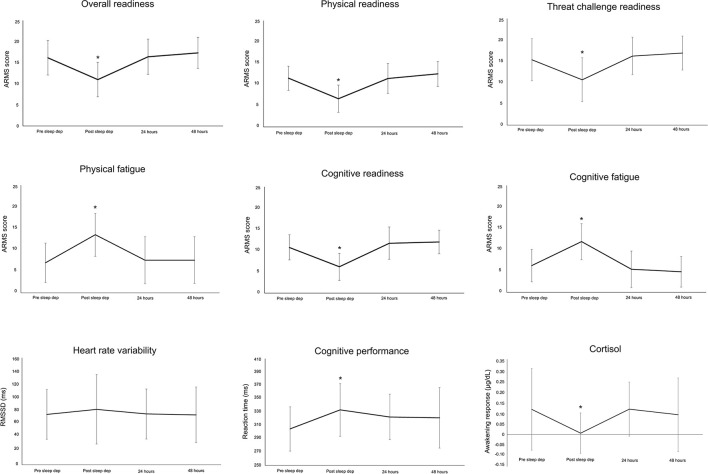
Mean and standard deviation for each subscale of the Acute Readiness Monitoring Scale (ARMS), heart rate variability (root mean square of successive differences between normal heartbeats, RMSSD), cortisol, and cognitive performance before (pre-sleep dep), immediately after (post-sleep dep), and 24 and 48 h post the sleep deprivation protocol. *p < 0.05 to pre-sleep dep measure.

Each ARMS subscale changed in response to sleep deprivation[Overall Readiness: LMEM: *F*_(3, 84)_ = 25.23, *p* < 0.001; Physical Readiness: LMEM: *F*_(3, 84)_ = 29.37, *p* < 0.001; Physical Fatigue: LMEM: *F*_(3, 84)_ = 19.58, *p* < 0.001; Cognitive Readiness: LMEM: *F*_(3, 84)_ = 29.40, *p* < 0.001; Cognitive Fatigue: LMEM: *F*_(3, 85)_ = 27.30, *p* < 0.001; Threat Challenge Readiness: LMEM: *F*_(3, 86)_ = 16.69, *p* < 0.001]. Relative to baseline (pre-sleep deprivation), Overall Readiness, Physical Readiness, Cognitive Readiness, and Threat Challenge Readiness was reduced immediately post-sleep deprivation (*post-hoc* for all: *p* < 0.001; Figure 4), while Physical Fatigue and Cognitive Fatigue increased (*post-hoc* for all: *p* < 0.001, Figure 4). All scores on the ARMS subscales returned to baseline 24- and 48-h after sleep deprivation (*post-hoc* for all comparisons between baseline and 24- and 48-h post: *p* > 0.33, Figure 4). Similarly, awakening responses in cortisol and cognitive performance changed in response to sleep deprivation [cortisol: LMEM: *F*_(3, 81)_ = 4.69, *p* = 0.005; cognitive performance: LMEM: *F*_(3, 84)_ = 6.73, *p* < 0.001]. Relative to baseline (pre-sleep deprivation), lower awakening responses in cortisol and reduced cognitive performance (slower reaction time) was observed immediately post-sleep deprivation (*post-hoc* for all: *p* < 0.02; Figure 4), with both these outcomes returning to baseline 24/48 h post-sleep deprivation (*post-hoc* for all comparisons between baseline and 24- and 48-h post: *p* > 0.06, Figure 4). No change in HRV (RMSSD) was observed in response to sleep deprivation [LMEM: *F*_(3, 79)_ = 1.16, *p* = 0.33].

### The Relationship Between the ARMS and Biological Indicators of Readiness

No relationship was observed between the ARMS subscales and HRV (RMSSD) (Table 2). There was a relationship between each ARMS subscale and cortisol (Table 2). Lower scores on Overall Readiness, Physical Readiness, and Threat Challenge Readiness were moderately correlated with lower awakening responses in cortisol (Table 2). A similar relationship existed for Cognitive Readiness, but the strength of this correlation was weak (correlation coefficient < 0.3). Higher scores on Cognitive Fatigue and Physical Fatigue were weakly correlated with lower awakening responses in cortisol (Table 2).

**Table 2:** Repeated measures correlations between all variables, including each subscale of the ARMS, heart rate variability, cortisol, and cognitive performance.

	**Overall readiness**	**Cognitive readiness**	**Physical readiness**	**Cognitive fatigue**	**Physical fatigue**	**Threat challenge readiness**	**RMSSD**	**Cortisol response**	**PVT RT**
Overall readiness	–	0.860^****^	0.861^****^	−0.848^****^	−0.706^****^	0.880^****^	0.116	0.422^****^	−0.192
Cognitive readiness		–	0.882^****^	−0.874^****^	−0.713^****^	0.883^****^	0.089	0.319^***^	−0.265^*^
Physical readiness			–	−0.828^****^	−0.751^****^	0.804^****^	0.071	0.362^****^	−0.160
Cognitive fatigue				–	0.735^****^	−0.815^****^	−0.070	−0.324^***^	0.285^***^
Physical fatigue					–	−0.707^****^	−0.048	−0.292^**^	0.291^***^
Threat challenge readiness						–	0.133	0.340^***^	−0.189
RMSSD							–	0.035	0.156
Cortisol response								–	−0.078
PVT RT									–

*RMSSD, root mean square of successive differences between normal heartbeats; PVT, psychomotor vigilance test; RT, reaction time*

* *<0.05,*

** *<0.01,*

*** *<0.005,*

**** *<0.001*.

### The Relationship Between the ARMS and Cognitive Performance

**Figure 5:**
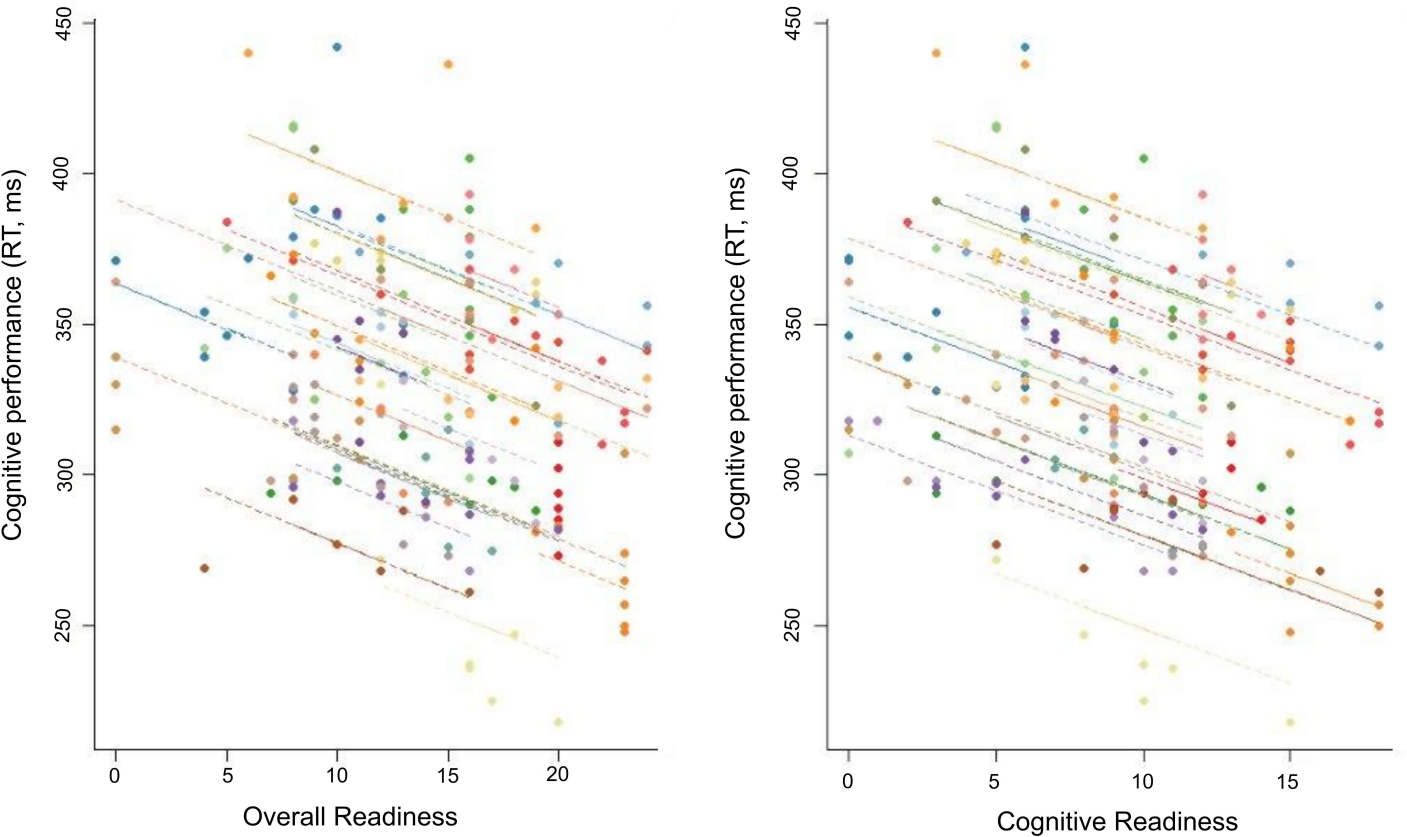
Repeated-measures correlation between cognitive performance and two subscales (Overall Readiness, Cognitive Readiness) of the Acute Readiness Monitoring Scale (ARMS) for the overnight assessments (from 20:00 to 08:00 h).

Lower scores on Cognitive Readiness and higher scores on Cognitive Fatigue and Physical Fatigue were correlated with slower reaction time (Table 2). No relationship was observed between any other ARMS subscale and cognitive performance (Table 2). *Post-hoc* analyses of the overnight assessments further supported a relationship between the ARMS and cognitive performance. That is, lower scores on Overall Readiness (CC: −0.53, *p* < 0.01), Cognitive Readiness (CC: −0.54, *p* < 0.01), Physical Readiness (CC: −0.47, *p* < 0.01), and Threat Challenge Readiness (CC: −0.50, *p* < 0.01) and higher scores on Cognitive Fatigue (CC: 0.50, *p* < 0.01) and Physical Fatigue (CC: 0.56, *p* < 0.01) strongly correlated with slower reaction time. Two examples of this relationship are illustrated in Figure 5.

### Exploratory Analysis Investigating the Predictive Value of the ARMS and Biological Markers of Readiness to Cognitive Performance

Given that Cognitive Readiness, Cognitive Fatigue, and Physical Fatigue subscales were significantly correlated with cognitive performance over the course of the sleep deprivation protocol (pre-sleep deprivation, immediately post, 24/48 h post), these scales were input into the multivariate regression. As a single independent variable, all three ARMS scales were highly predictive of cognitive performance (Table 3, Model 1 for each). This model improved when HRV and cortisol were added. Higher AIC/BIC values were reported when each ARMS subscale was combined with HRV and cortisol (Model 4) than when each was input alone (Model 1) or when HRV and cortisol were input separately (Model 2 & 3; Table 3). Similarly, the explained variance in each model (marginal r-squared) tended to improve when HRV and cortisol were added, though HRV contributed more to the variance in each model than cortisol (Table 3).

**Table 3:** Multivariate regression models testing whether the ARMS subscales (Cognitive Readiness, Cognitive Fatigue, Physical Fatigue) predict cognitive performance with and without HRV and cortisol.

	**Estimate**	***P*** **-value**	**Conditional *r^**2**^***	**Marginal *r^**2**^***	**AIC**	**BIC**
**Model 1**						
Cognitive readiness	−2.08	0.004	0.541	0.044	1,128.0	1,139.0
**Model 2**						
Cognitive readiness	−2.21	0.003	0.522	0.145	1,063.6	1,077.0
RMSSD	−20.92	0.009				
**Model 3**						
Cognitive readiness	−2.11	0.007	0.548	0.043	1,103.8	1,117.5
Cortisol	3.74	0.86				
**Model 4**						
Cognitive readiness	−2.29	0.005	0.526	0.147	1,039.2	1,055.2
RMSSD	−21.35	0.009				
Cortisol	8.65	0.686				
**Model 1**						
Cognitive fatigue	1.50	0.012	0.571	0.033	1,130.5	1,141.5
**Model 2**						
Cognitive fatigue	1.75	0.006	0.536	0.123	1,065.0	1,078.5
RMSSD	−22.86	0.006				
**Model 3**						
Cognitive fatigue	1.45	0.026	0.573	0.030	1,106.6	1,120.3
Cortisol	0.49	0.982				
**Model 4**						
Cognitive fatigue	1.76	0.011	0.535	0.124	1,041.0	1,057.0
RMSSD	−23.43	0.006				
Cortisol	7.25	0.735				
**Model 1**						
Physical fatigue	1.39	0.011	0.577	0.040	1,130.7	1,141.6
**Model 2**						
Physical fatigue	1.57	0.007	0.551	0.123	1,065.6	1,079.0
RMSSD	−21.85	0.009				
**Model 3**						
Physical fatigue	1.69	0.005	0.614	0.054	1,104.1	1,117.7
Cortisol	2.16	0.915				
**Model 4**						
Physical fatigue	1.94	0.002	0.587	0.135	1,038.8	1,054.8
RMSSD	−22.40	0.008				
Cortisol	8.13	0.692				

*Dependent variable for all the above regressions: Cognitive performance (PVT reaction time); AIC, Akaike information criterion; BIC, Bayesian information criterion; RMSSD, root mean square of successive differences between normal heartbeats*.

## Discussion

This criterion validation study set out with three specific aims. Regarding the first, our data demonstrated that the ARMS is responsive to a 24-h sleep deprivation protocol, with all six of the deployed subscales altered immediately after sleep deprivation and returning to baseline 24/48 h post. This finding suggests the ARMS has utility in detecting acute fluctuations in an individual's perceived readiness to sleep deprivation. Regarding the second aim, our analysis demonstrated several associations between ARMS subscales and biological markers of readiness, specifically with awakening responses in cortisol, but not HRV. This finding supports the validity of the ARMS and suggests that it may be a useful proxy for biological markers of readiness. Finally, regarding the third aim, our analysis demonstrated that several ARMS subscales (i.e., Cognitive Readiness, Cognitive Fatigue, Physical Fatigue) predicted the change in cognitive task performance in response to sleep deprivation, suggesting that the ARMS may hold promise for monitoring and mitigating potential underperformance on tasks requiring attention and vigilance.

The finding that a psychometric scale can be responsive to acute sleep deprivation has been demonstrated previously (e.g., the Karolinska Sleepiness Scale—Kaida et al., 2006; Driver Impairment Scale—Jongen et al., 2015; Sleep Hygiene Scale—Murawski et al., 2019). Consistent with this research, the ARMS was responsive to a 24-h sleep deprivation protocol, detecting fluctuations in an individual's perceived readiness. The ARMS is a brief, easily interpreted measure of perceived readiness for any key task or role, in the immediate future, and so demonstrating its responsiveness to sleep deprivation highlights the potential of using the ARMS to make meaningful inferences about individual- and group-readiness in field settings (i.e., by simply administering with pen-and-paper). Given the well-recognised impacts of sleep deprivation on cognitive, physical, and sports performance (Nindl et al., 2002; Alhola and Polo-Kantola, 2007; Roca et al., 2012; Samuels, 2012; Parker and Parker, 2017; Grandou et al., 2019; Beckner et al., 2021), the ability to potentially pre-empt and mitigate these effects (by monitoring perceptual readiness), may facilitate improved outcomes for performance and training, although this suggestion is speculative and requires confirmation using larger samples and longitudinal study designs.

The finding that ARMS subscales demonstrated correlations with cortisol, but not HRV, offers support for the concurrent validity of the instrument. While seeking concurrent validation against physiological markers is increasingly called for (Lee, 2012), very few studies have attempted to do so, and indeed even fewer demonstrate such an association (Hellhammer et al., 2009). Further, the scales that have been linked to variations in cortisol response were indicators of more chronically stable trait indices (e.g., perceived stress management skills –Wirtz et al., 2013; stress overload—Amirkhan et al., 2015), not acute measures. As such, the ARMS may offer strong potential for a method of detecting underlying physiological changes in readiness that does not depend on sophisticated sampling, storage, and analysis of biological samples.

The reason why HRV (RMSSD) did not relate to ARMS subscales is not known, but may be related to the high intra-individual variability observed. Previous research has demonstrated that while stress (from training) can decrease vagal tone (Iellamo et al., 2002), it can also lead to increased intra-individual variance of HRV parameters (Schmitt et al., 2013). Further, while previous studies have demonstrated decreases in HRV (RMSSD) following 24-h of sleep deprivation (Chen et al., 2013; Morales et al., 2019), this response is not always consistent (Pagani et al., 2009; Quintana et al., 2017). Alternatively, fatigue from overtraining can lead to a bell curve trend in HRV data–i.e., a non-linear relationship (Le Meur et al., 2013), undermining the linear analysis conducted in the present study. In this case, ARMS appeared to measure something different to HRV.

Notably, this study demonstrated a relationship between several ARMS subscales and PVT performance. The PVT task used in this study has relevance for sporting and military job roles, drawing on underlying capacities for sustained attention and response/processing times (Dinges and Kribbs, 1991; Warm et al., 2008; Jones et al., 2018). The ability to anticipate diminished performance in tasks that draw on these capacities may help in the management and planning of individual and/or group activities. For example, the assessment of perceived readiness prior to engaging in a sports event may provide valuable information on those individuals at risk of underperforming. Similarly, perceived readiness scores may be used to detect those at risk of dropout and/or course failure following extended periods of high training loads in military personnel. We note that there are, of course, many other domains of cognitive performance that were not assessed in the present study, such as working memory, response inhibition and task-switching, which warrant further validation with the ARMS subscales.

In the current study, the ARMS subscales were more predictive of PVT performance than the biological markers of readiness (cortisol/HRV showed no relationship). This suggests that perceptual measures of readiness may hold better utility, than typical biological markers, in monitoring and mitigating underperformance on tasks requiring attention and vigilance. However, it should be noted, that while the biological indicators of readiness (cortisol/HRV) did not predict cognitive performance alone, when combined with perceptual measures of readiness (ARMS subscale), they seem to be important in predicting performance (as seen by the improvement in model fit and explained variance, although this was predominantly improved with the addition of HRV and not cortisol). While this finding may have widespread implications for the design and implementation of protocols used to measure an individual's readiness, future work utilising larger samples are needed to confirm the interaction between perceptual (ARMS) and biological indicators of readiness in predicting cognitive performance. Such samples would allow for the investigation of potential mediating effects (MacKinnon et al., 2007) that may provide further insight into the nature of readiness.

### Future Directions

In light of the current findings, the ARMS may offer potential for implementation in day-to-day monitoring of performance readiness. Future research should continue exploring these potential associations by investigating different facets of cognitive performance, and by recruiting larger samples to more accurately quantify and model the relationships between perceptual and biological markers of readiness in predicting task performance. Likewise, different aspects of performance should be explored, such as physically demanding tasks, team-working tasks and communication. We note that in this study, we did not examine the validity of the ARMS subscales for “skills-training readiness;” “group-team readiness;” and “equipment readiness”—which warrant further testing. There is also an important consideration to be made in researching and optimising the implementation of ARMS in day-to-day practise and whether to encode the ARMS into a technology such as a digital Human Performance Management System, vs. paper-and-pen implementation. Further, the way that information is shared with, and used by, supporting staff will be critical, as the sense that such information may affect individual's careers may lead to biassed responding patterns that undermine the instruments utility.

### Limitations

Despite a robust approach to data collection and analysis, this study is not without limitations. Data were obtained from a small sample of young university students (mean age 23 ± 4 years), whose demographic characteristics likely reflect many sporting and military memberships e.g., see reference (Le Menestrel and Kizer, 2019). In this regard we were able to balance the need for control and relatively intrusive measurement approach against the need for large-scale real-world evaluation, but clearly future studies should extend to these other contexts: to either add statistical power, assess in more ecologically valid settings, or both. In some respects, sleep deprivation may appear to be a significant, disproportionate impost, relative to regular day-to-day hassles. Nevertheless, we would argue that a single night of sleep deprivation is comparable to shift work, long distance travel or accumulated daily stress faced in a range of settings.

Strict monitoring of participants was not possible during the sleep deprivation protocol, so some participants may have fallen or at least dozed asleep during the night. However, our accelerometer monitoring suggested participants were adequately active and remained awake, or at least did substantially disrupt their sleep. Likewise, visual inspection of the overnight assessments suggest that dozing was not a major problem, as subjective sleepiness increased and declines in task performance and ARMS scores were observed by most participants. A 24-h sleep deprivation protocol was used to manipulate an individual's perceived readiness. Further research is required to determine whether the findings of the present study translate to other manipulations of readiness over a longer time-period (e.g., fluctuations in day-to-day physical and cognitive workloads). It is possible that the 24-h sleep deprivation protocol used in the present study did not induce enough physiological stress to elicit changes in HRV. This may be related to the sample recruited. Participant were university students with average sleep durations at baseline that were lower than normal (6.4 hrs), suggesting participants may be familiar with periods of limited sleep. Further, behaviours prior to the night of sleep deprivation were not controlled, which may have influenced their physiological response to sleep deprivation (e.g., not exercising as much the day before or consuming more caffeine than usual). Lastly, multiplicity adjustments were not made for the correlational and regression analyses, increasing the likelihood of a Type 1 error. Nevertheless, this was an exploratory study for the purpose of continuing the development of a new and innovative monitoring instrument. The current paper offers insights that assist confirmatory research designs in the future.

## Conclusions

This study demonstrated that the ARMS is responsive to the effects of acute sleep deprivation while also relating to associated changes in awakening responses in cortisol and cognitive task performance. These data suggest that the ARMS may hold practical utility in detecting, or screening for, the wide range of deleterious effects caused by sleep deprivation; may constitute a quick, cheap and easily interpreted alternative to intrusive and technologically challenging physiological measures of readiness; and may be used to monitor or mitigate potential underperformance on tasks requiring attention and vigilance.

## Data Availability Statement

The original contributions presented in the study are included in the article figures and tables, further inquiries can be directed to the corresponding author.

## Ethics Statement

This study received ethical approval by the University of Canberra Human Research Ethics Committee. The participants provided their written informed consent to participate in this study.

## Author's Note

This paper captures the process followed in developing a psychometrically sound instrument for daily (or frequent) monitoring of Australian military personnel, commissioned at the request of Australian Army, with a focus on applied utility and suitability for immediate practical implementation into existing systems and practises.

## Author Contributions

SS, RK, AF, KM, AM, and BR contributed to the conception and design of the work. SS was responsible for data collection. All authors were involved in the analysis, writing, editing of the manuscript, and approved the manuscript prior to submission.

## Funding

This research was funded by Defence Science and Technology Group, in collaboration with the Australian Army.

## Conflict of Interest

The authors declare that the research was conducted in the absence of any commercial or financial relationships that could be construed as a potential conflict of interest.

## Publisher's Note

All claims expressed in this article are solely those of the authors and do not necessarily represent those of their affiliated organizations, or those of the publisher, the editors and the reviewers. Any product that may be evaluated in this article, or claim that may be made by its manufacturer, is not guaranteed or endorsed by the publisher.
